# Considerations regarding the concurrent validity of the automatic cashier test for information processing speed

**DOI:** 10.1590/1980-5764-DN-2026-0529

**Published:** 2026-07-20

**Authors:** Bhoomeeka Jayramdass, Isheeka Lohana

**Affiliations:** 1 Liaquat University of Medical and Health Sciences, Hyderabad, Sindh, Pakistan.; 2 Indus Medical College, Tando Muhammad Khan, Sindh, Pakistan.

Dear Editor,

We read with great interest the article by Zavala et al. on the concurrent validity of the automatic cashier test (AC-T) for assessing information processing speed^1^. The authors should be commended for proposing a practical screening tool that may reduce graphomotor demands and offer a simpler alternative for older adults. Given the importance of rapid cognitive screening in clinical settings, this study addresses a relevant and timely topic.

However, a number of the study's findings should be interpreted cautiously. One concern relates to the use of the term "validation," which may be stronger than the reported results support. Although the association between AC-T and the digit symbol test (DS-T) was statistically significant, the overall coefficient of determination was only R²=0.40, with subgroup values ranging from 0.14 to 0.58^1^. The authors also acknowledged that the AC-T still requires adjustment, as these values fall below the suggested range. Therefore, rather than providing conclusive validation, the results might be better understood as preliminary evidence of concurrent association. Since processing speed is a broad cognitive construct, significant association alone may not be sufficient to establish robust validity^2^.

Another important consideration is that the AC-T and DS-T may not assess identical constructs. According to the article, the AC-T depends on simpler motor selection and familiar alphabetic stimuli, while the DS-T incorporates fixed-time performance, symbol learning, recall, and graphomotor execution^1^. Prior studies have demonstrated that performance on tasks related to processing speed may also be influenced by working memory, attention, and visuomotor coordination^3-6^. Thus, the modest relationship observed between the two tests may partly reflect variations in cognitive and motor demands rather than only limited performance of the AC-T itself.

Furthermore, some methodological aspects deserve more clarification. The initial sample of 139 participants was reduced to 130 after influential-point analysis^1^. Although the exclusion of extreme value may be statistically justified, more detail regarding how these points were identified and whether the primary conclusions remained consistent before and after exclusion would improve transparency. The generalizability of the findings appears limited, as the sample consisted of Spanish-speaking adults aged 55–84 years from a single metropolitan area, with most participants having primary or secondary education^1^. Since educational level can affect cognitive performance and cognitive reserve^2,7^, these findings should be generalized cautiously to other populations.

In conclusion, this study offers an innovative contribution toward simplified information processing speed screening. However, a broader psychometric evaluation, clearer reporting of influential-point handling, and assessment in more diverse populations may be necessary before the AC-T can be considered fully validated for routine use.

## Data Availability

No new data were generated or analyzed in this study.
